# Development of a Japanese Version of the Daily Record of Severity of Problems for Diagnosing Premenstrual Syndrome

**DOI:** 10.1089/whr.2019.0004

**Published:** 2020-01-20

**Authors:** Yumie Ikeda, Miho Egawa, Kazuko Hiyoshi, Tsukasa Ueno, Keita Ueda, Carl B. Becker, Yoshimitsu Takahashi, Takeo Nakayama, Masaki Mandai

**Affiliations:** ^1^ Department of Obstetrics and Gynecology, Kyoto University Hospital, Kyoto, Japan.; ^2^ Department of Health Informatics, Kyoto University School of Public Health, Kyoto, Japan.; ^3^ Faculty of Nursing, Doshisha Women's College of Liberal Arts, Kyotanabe, Japan.; ^4^ Department of Neuropsychiatry, Graduate School of Medicine, Kyoto University, Kyoto, Japan.; ^5^ Science Policy Unit, Kyoto University School of Medicine, Kyoto, Japan.

**Keywords:** Japanese, daily record of severity of problems, premenstrual syndrome

## Abstract

***Background:*** Premenstrual syndrome (PMS) causes physical and mental symptoms in women during the luteal phase of the menstruation cycle. To confirm the relationship between symptoms and the menstruation cycle, daily symptom records are essential for diagnosing PMS. The daily record of severity of problems (DRSP) is currently the most validated tool for tracking symptoms to confirm and scale the severity of PMS, but there has been no validated Japanese version of this instrument. We developed a Japanese version of the DRSP and assessed its psychometric properties.

***Methods:*** A Japanese version of the DRSP was developed following the translation guidelines of the latest Patient-Reported Outcomes Consortium. We conducted a computational psychometric study among 119 women, all of whom completed the Japanese version of the DRSP, the Center for Epidemiologic Studies Depression Scale (CES-D), the Premenstrual Dysphoric Disorder (PMDD) scale, and a Numerical Rating Scale (NRS) for general health status. Each was filled out twice, 2 weeks apart, so that one set was completed in the luteal phase.

***Results:*** The Japanese version of the DRSP was developed with translation and cultural adaptation. The internal consistency coefficient for the total score was 0.93. The DRSP in the luteal phase correlated highly with the CES-D, PMDD scale, and NRS for general health status.

***Conclusions:*** Our Japanese version of the DRSP, developed as a PMS/PMDD diagnostic tool, was shown to provide substantial validity and reliability to rate premenstrual symptoms for Japanese women.

## Introduction

Premenstrual syndrome (PMS) causes physical and mental symptoms in women during the luteal phase of the menstruation cycle. Approximately 70%–90% of menstruating women experience some premenstrual symptoms,^1^ and 20%–40% experience troublesome PMS. Between 1% and 8% of women have premenstrual dysphoric disorder (PMDD), the most severe form of PMS, which interferes with daily work, school, social life, and relationships.^1–3^

The American College of Obstetrics and Gynecology (ACOG) has defined diagnostic criteria for PMS that require patients keep prospective daily records to determine whether symptoms worsen cyclically, but it has not specified a recording tool.^3^ Prospective recording tools include the daily record of severity of problems (DRSP), the premenstrual records of impact and severity of menstruation (PRISM), the calendar of premenstrual experiences (COPE), and the daily symptom report (DSR)^4^; recently, the British Royal College of Obstetrics and Gynecology (RCOG) recommended using the DRSP, currently the most commonly recommended tool for tracking premenstrual symptoms.^5^ The DRSP was created in 1997 based on the diagnostic criteria for PMDD of the *Diagnostic and Statistical Manual of Mental Disorders, Fourth Edition* (DSM-IV).^6^ The DSM-5, released in 2013, was essentially the same criteria for diagnosis.^7^ Therefore, the DRSP is still most widely recommended. The utility and validity of the English version of the DRSP in measuring the severity and fluctuations of PMS have been established.^8^

Although some retrospective studies have estimated that PMS affects almost one-fifth of Japanese women,^9^ Japan lacks any validated diagnostic tool for PMS. Many epidemiological studies of PMS in Japan have used retrospective questionnaires^9–13^ that have shown little correspondence with diagnoses based on prospective recording.^14,15^ Moreover, no Japanese scale measured the severity of PMS. Just as we were conducting our own work on this field, one Japanese version of the DRSP has been reported,^16^ substantially advancing the possibilities for PMS research in Japan. However, the DSRP includes a range of psychological elements demanding great care and cultural sensitivity in any translation. Only in 2018, the Patient-Reported Outcomes (PRO) Consortium (formed by the Critical Path Institute [C-Path] in cooperation with the U.S. FDA Center for Drug Evaluation and Research and the pharmaceutical industry) updated their guide to enable different translation companies to share standard procedures for translation and cultural adaptation (TCA).^17^ These new guidelines require 12 carefully defined steps to achieve an unprecedented level of precision in medical translation, including reconciliation of plural parallel fore translations and back translations, cognitive interviewing, and incorporation of interview feedback into the final translation. The DSRP-J that has just appeared is a welcome new focus of attention on this field, but it fails to explicitly incorporate comparison and reconciliation of plural translations, much less interviews about their psychological impact on the types of respondents to be studied. Convinced of the importance of not only medically precise but also culturally and psychologically sensitive wording, we based our new translation on PRO Consortium guidelines, and statistically confirmed its validity and reliability as a diagnostic scale for PMS.

## Methods

### Daily record of severity of problems

The DRSP comprises 21 items describing premenstrual symptoms and 3 items concerning the dysfunction in daily life caused by these symptoms.^18^ Throughout the menstrual cycle, all 24 items are ranked daily from 1 (not at all) to 6 (extreme). The DRSP contains three clinically determined subscales: depressive symptoms (felt depressed, felt hopeless, felt worthless or guilty, slept more, trouble sleeping, and felt overwhelmed); physical symptoms (breast tenderness, bloating, headache, and joint or muscle pain); and anger/irritability (anger/irritability and personal conflicts). A total DRSP score of <50 at the onset of menstruation indicates the absence of PMS.^18^

### TCA of the DRSP

In 2005, principles of good practice for the TCA of PRO measures was published by the International Society for Pharmacoeconomics and Outcomes Research (ISPOR).^19^ In 2018, the PRO Consortium (formed by the Critical Path Institute [C-Path] in cooperation with the U.S. FDA Center for Drug Evaluation and Research and the pharmaceutical industry) updated this guide to enable different translation companies to share standard procedures for TCA.^17^ The new guide did not change the principles of TCA, but it details how to perform each step and how to adapt the TCA process to different situations worldwide.^17^

The Japanese version of the DRSP was developed based on the 2018 PRO Consortium consensus guide for translating and culturally adapting PRO into other languages, which constitutes a 12-step process (Supplementary Appendix Table SA1). We followed the 12 steps to develop the Japanese version of the DRSP, including conducting cognitive interviews (step 9) of 8 PMDD outpatients at Kyoto University Hospital.

### Participants

Our research was intended to examine the reliability and validity of the DRSP for measuring PMS in Japanese. We recruited study participants from October to December 2018 from students at the Public School of Kyoto University and Doshisha Women's College of Liberal Arts, and from staff members at Kyoto University Hospital. The convenience sample included 119 women (median age, range = 31.8, 20–51 years) who reported regular menstruation and who were not taking any hormonal agents.

Participants were handed two sets of questionnaires and asked to answer them at home and return them by mail. One set was to be completed on the day they received it, and the other to be completed 2 weeks later. Since PMS symptoms commonly express in the latter half of the menstrual cycle, this survey only used data recorded 2 weeks or more after the beginning of the cycle. If a participant completed the forms twice in the luteal phase, only the second set was analyzed. Subjects who failed to return the second set of questionnaires were recalled by e-mail; those who did not complete both questionnaires were excluded.

### Measurements

Each questionnaire contained the Japanese version of the DRSP, the Center for Epidemiologic Studies Depression Scale (CES-D),^20^ the PMDD scale,^21^ and the Numerical Rating Scale (NRS) for general health status.

The CES-D is a self-reported 20-item questionnaire used to detect depression. The score ranges from 0 to 60, with a higher score indicating more severe depression; scores of >19 are considered borderline to full depression. The Japanese version of this tool was created in 1985, and has since been established as a valid and reliable depression scale in many studies.^20^

The PMDD scale used the Japanese version of the DSM-5 diagnostic criteria for PMDD. These criteria include 11 items regarding physical and emotional symptoms that emerge 1–2 weeks before menstruation and disappear after onset of menstruation. Participants answer these questions retrospectively based on recollection of their symptoms. The remaining three items ask how the mentioned symptoms interfered with daily life. All items are ranked from 1 to 4, with higher scores indicating more severe symptoms. Previous studies have confirmed the validity and reliability of the Japanese PMDD scale.^21,22^

The NRS used in this study was an 11-point scale used to assess general health status on the day the scale was completed, from 0 (the worst of your life) to 10 (the best of your life).

### Reliability and validity

To assess the internal consistency of the Japanese version of the DRSP, we calculated Cronbach's α's for the total DRSP and for each of its three subscales. Cronbach's α's of 0.7–0.8 are considered satisfactory.

To confirm criteria validity, we used the CES-D, PMDD scale, and NRS for general health status. We calculated Pearson's coefficients, with *p* values, between the total score of the 21 symptom-related items in the DRSP Japanese version and the mentioned three measures. We also calculated correlations between the depressive symptoms subscales in the DRSP and CES-D. Two sided tests were done with a significance level of 0.001. Statistical analyses were performed using Stata version 15.1.

#### Ethical considerations

The Kyoto University Ethics Committee approved this study. The aims and procedures of this study were explained to all participants, and they were permitted to withdraw from the study at any time. Written informed consent was obtained from each participant.

## Results

### Development of a Japanese version of the DRSP and cultural adaptation

After obtaining the original DRSP authors' permission^18^ to translate the DRSP into Japanese, we first developed an Item Definition Table that described “the concepts being measured along with translation alternatives.” The second step involved forward translations of the DRSP, performed independently by a Japanese doctor of gynecology and by a professional nurse; in the third step, yet another Japanese doctor of gynecology joined the team in their comparison and tentative reconciliation of these two translations. We then took this tentative translation to two native English scholars intimately familiar with Japanese psychology and culture, for their double-blinded translation back into English.

Next, we convened a team including two more clinical psychiatrists to discuss the discrepancies and difficulties emerging in the back translation based on their clinical experience with PMS and PMDD patients. In step 7, >10 native Japanese speakers proofread our Japanese DRSP and independently affirmed that no revisions were needed. In step 8, we interviewed eight outpatients with PMS, including five with PMDD seeing psychiatrists at Kyoto University Hospital, to evaluate their reactions and responses to our Japanese DRSP.

Their median time to complete the DRSP for 1 day's symptoms was 2 minutes 5 seconds. Three of the patients had no questions or comments about the tool; five had opinions or comments, presented in Table 1. Based on our discussion of these opinions, we concluded that no linguistic changes were needed. In step 9, all members of the translation team discussed the results of the cognitive interviews, and in step 10, we developed a final version of the Japanese DRSP. In steps 11 and 12, this Japanese DRSP was reported to all the stakeholders and was archived. (Supplementary Appendix Tables SAT2 and SAT3).

**Table 1.** Summary of Premenstrual Syndrome/Premenstrual Dysphoric Disorder Patient Cognitive Interviews (*n* = 8)

Characteristics of participants (median, range)	
Age (years)	42 (21–43)
Time to answer (minutes)	2’00 (1’08–4’50)
Feedback	
Symptoms	I cannot understand feeling “on edge” because I do not have that symptom.
	It is strange that this questionnaire has no question on peripheral edema, because I have that symptom.
	It is difficult to distinguish between breast tenderness and breast swelling.
Instruction	Instructions say to answer about “today,” but on item 15 (had trouble getting to sleep or staying asleep), I have to answer about last night.
	Answering about today is tough because my symptoms change during the day.

### Reliability and validity

#### Demographic profile of participants

**FIG. 1.**
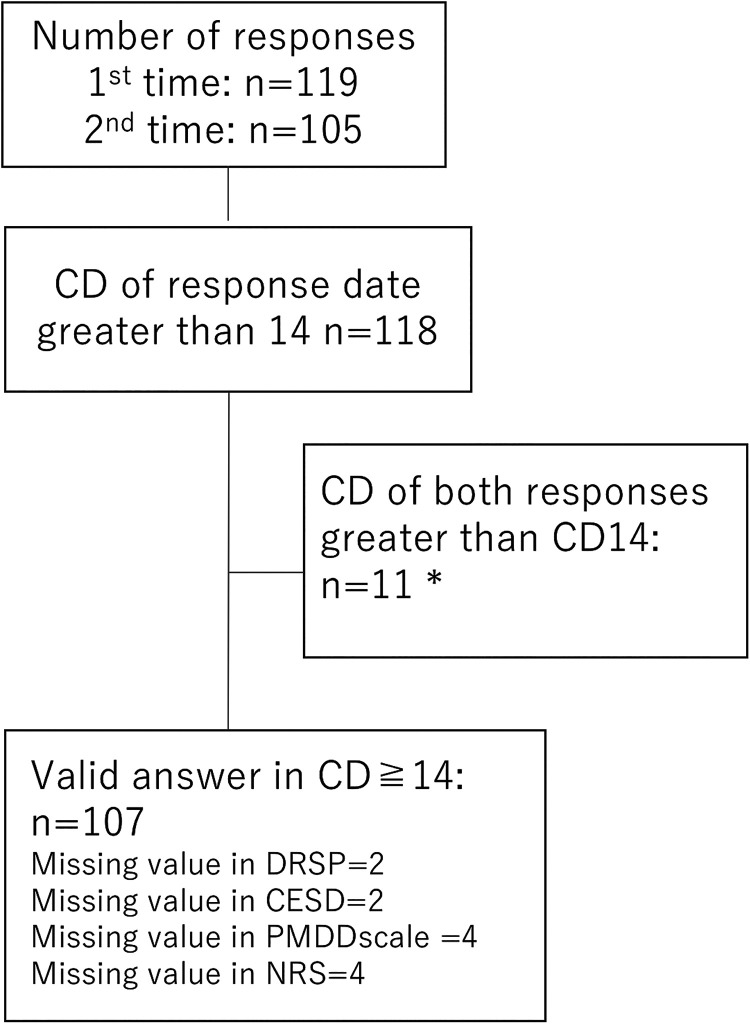
Number of participants. *Remove the earlier answer. CD = cycle date; CES-D = Center for Epidemiologic Studies Depression Scale; DRSP = daily record of severity of problems; NRS = Numerical Rating Scale; PMDD = Premenstrual Dysphoric Disorder.

Of 119 participants, 118 completed questionnaire sets 14 or more days after the start of menstruation. In 11 cases, the participants' two response sets were *both* in the period beginning 2 weeks after the onset of menstruation, so we excluded their earlier response sets, leaving a total of 107 responses within the period of concern Fig 1. Respondents' median age was 30 years (ranging from 21 to 46), and their median date of response was 21 (15–35) days into their menstrual cycle (Table 2). Their median total score of the 24 items on the Japanese DRSP was 34 (24–85), with 23 scoring >50 on the Japanese version of DRSP and >19 on the CES-D.

**Table 2.** Participants' Demographic Data

Demographics (median, IQR)	
Age	30 (26–36)
Cycle day on the response date	21(17–26)
Total score of Japanese version of the DRSP (missing value *n* = 2)	34 (24–85)
Total score of CES-D (missing value *n* = 2)	9 (5–16)
Total score of PMDD (missing value *n* = 4)	30 (23–41)
NRS for general health (missing value *n* = 4)	7 (5–8)

CES-D, Center for Epidemiologic Studies Depression Scale; DRSP, daily record of severity of problems; NRS, Numerical Rating Scale; PMDD, Premenstrual Dysphoric Disorder.

#### Internal consistency

Cronbach's α for the total DRSP score was 0.93 for the luteal phase. The internal consistencies of depression symptoms, physical symptoms, and anger and irritability were 0.86, 0.68, and 0.80, respectively (Table 3).

**Table 3.** Internal Consistency (Cronbach's α) for Summary Scores of the Daily Records of Severity of Problems in Luteal Phase

	Item number	Chronbach's α
Total scores	Item 1–21	0.93
Depressive symptoms	Item 1.2.3.14.15.16	0.86
Physical symptoms	Item 18.19.20.21	0.68
Anger/irritability	Item 7.8.	0.80

#### Concurrent validity

Patients' total DRSP during the late luteal phase significantly correlated with their CES-D score, PMDD scale score, and NRS score (Table 4). The CES-D score correlated more strongly with the summary score of the depressive symptoms subscale of the DRSP than with the total DRSP score.

**Table 4.** Correlation of Daily Records of Severity of Problems Summary Scores with Other Measures of Severity

	PMDD Scale Total	CES-D Total	NRS
Total	0.57^*^	0.71^*^	−0.48^*^
Depressive Symptoms	0.52^*^	0.74^*^	−0.49^*^
Physical Symptoms	0.27	0.24	−0.23
Anger/Irritability	0.43^*^	0.41^*^	−0.29

^*^ *p* < 0.001

PMDD Scale, Premenstrual Dysphoric Disorder Scale; CES-D, Center for Epidemiologic Studies Depression Scale; NRS, Numerical Rating Scale.

## Discussion

This is the first documented translation of any medical questionnaire in Japan following the new TCA guidelines of the PRO Consortium. The Japanese DRSP was developed following all the steps in the PRO Consortium's translation guide. Following the TCA steps, we were able to discuss the uniqueness of expressions that Japanese patients often use in clinical settings, because our translating team included several clinicians who treat many PMS/PMDD patients.

Another point of discussion was verb tense. In the meeting of all the translation members, which included both native Japanese and English speakers, we found that in Japanese, current symptoms are more readily expressed in the present tense than the past tense. We decided to adopt the past tense following original English expression, but changed the instruction guide sentence in the DRSP sheet so that participants would understand they could record their symptoms at night while looking back on their whole day. As a result, we needed no major changes to the Japanese DRSP after interviewing the PMS/PMDD patients. We had no difficulty implementing the TCA process using the new translation guide, but found it quite useful.

We confirmed the reliability and validity of the Japanese version of the DRSP in 119 Japanese women whose PMS ranged from asymptomatic to severe. The internal consistency of our Japanese DRSP was quite high, and the concurrent validity with the CES-D, PMDD scale, and NRS for general health status was confirmed. The relatively low Cronbach's α for the physical symptoms summary score was considered to be due to variations in both the cycle dates on which data were acquired and to the severity of the participants' premenstrual symptoms. With regard to concurrent validity, the correlation with the CES-D was higher for the depressive symptoms subscale than for the total DRSP, indicating that the DRSP does not assess depression, but more specifically evaluates PMS/PMDD.

### Limitations and discussion

Our translation process overcame many limitations implicit in less rigorous methods. Our use of a 2-week cutoff to approximate the beginning of the luteal phase might be disputed, but we did confirm the regularity of menstruation of all our participants in the recruiting process. On the positive side, our recruitment of participants from a variety of medical facilities provided a broad range of ages and social backgrounds.

Our statistical confirmations include the selection bias inherent in using a convenience sample of outpatients, and some might criticize our sample size of 119. Despite our relatively small sample size, the fact that we were still able to derive a high Cronbach's α as well as highly significant correlations with CES-D, PMDD, and NRS scores confirms both the internal consistency and external validity of our Japanese version of the DRSP.

To perfect this analysis, we need to collect Japanese version of DRSP data with more precise cycle dates by taking continuous symptoms recording throughout menstruation cycle. If we can get patients to continuously monitor their daily symptoms, our Japanese version of the DRSP should prove a highly reliable scale for premenstrual symptoms.

In conclusion, our Japanese translation of the DRSP provides for the first time a measurement tool precisely comparable with the English original on both linguistic and statistical grounds. In addition, our Japanese version of the DRSP displays baseline psychometric properties to measure premenstrual symptoms for Japanese women. Additional studies are expected to confirm its more adequate reliability and validity.

## Supplementary Material

Supplemental data

Supplemental data

Supplemental data

## Supplementary Material

Supplementary Appendix Table SA1

Supplementary Appendix Table SA2

Supplementary Appendix Table SA3

## Abbreviations Used

ACOG: American College of Obstetrics and Gynecology
CES-D: Center for Epidemiologic Studies Depression Scale
COPE: Calendar of Premenstrual Experiences
C-Path: Critical Path Institute
DRSP: daily record of severity of problems
DSM-IV: *Diagnostic and Statistical Manual of Mental Disorders, Fourth Edition*
DSR: daily symptom report
ISPOR: International Society for Pharmacoeconomics and Outcomes Research
NRS: Numerical Rating Scale
PMDD: Premenstrual Dysphoric Disorder
PMS: premenstrual syndrome
PRISM: Premenstrual Records of Impact and Severity of Menstruation
PRO: Patient-Reported Outcomes
RCOG: Royal College of Obstetrics and Gynecology
TCA: translation and cultural adaptation

## References

[1] European Medicine Agency. Guideline on the treatment of premenstrual dysphoric disorder (PMDD) 2011. https://www.ema.europa.eu/en/documents/scientific-guideline/adopted-guideline-treatment-premenstrual-dysphoric-disorder-pmdd_en.pdf viewed in January 9th, 2020

[2] Gehlert S, Song IH, Chang CH, Hartlage SA The prevalence of premenstrual dysphoric disorder in a randomly selected group of urban and rural women. Psychol Med 2009;39:129–136 18366818 10.1017/S003329170800322X PMC2752820

[3] The American College of Obstetrician and Gynecologist. Guidelines for women's health care a resource manual, 4th ed. Washington, DC Editorial Committee for Guidelines for Women's Health Care, 2014

[4] Steiner M, Pearlstein T, Cohen LS, et al. Expert guidelines for the treatment of severe PMS, PMDD, and comorbidities: The role of SSRIs. J Womens Health (Larchmt) 2006;15:57–69 16417420 10.1089/jwh.2006.15.57

[5] Green L, O'Brien, N Panay, Craig M, on behalf of the Royal College of Obstetricians and Gynaecologists. Management of Premenstrual Syndrome: Green-top Guideline No. 48. BJOG 2017;124:e73–e105 27900828 10.1111/1471-0528.14260

[6] Yonkers KA, Halbreich U, Freeman E, et al. Symptomatic improvement of premenstrual dysphoric disorder with sertraline treatment. A randomized controlled trial. Sertraline Premenstrual Dysphoric Collaborative Study Group. JAMA 1997;278:983–988 9307345

[7] American Psychiatric Association. Diagnostic and Statistical Manual of Mental Disorders (DSM–5) 2013 Washington, DC American Psychiatric Association

[8] Yonkers KA, Cameron B, Gueorguieva R, Altemus M, Kornstein SG The influence of cyclic hormonal contraception on expression of premenstrual syndrome. J Womens Health (Larchmt) 2017;26:321–328 27854559 10.1089/jwh.2016.5941 PMC5397197

[9] Tadakawa M, Takeda T, Monma Y, Koga S, Yaegashi N The prevalence and risk factors of school absenteeism due to premenstrual disorders in Japanese high school students-a school-based cross-sectional study. Biopsychosoc Med 2016;10:13 27118993 10.1186/s13030-016-0067-3 PMC4845482

[10] Takeda T, Imoto Y, Nagasawa H, Muroya M, Shiina M Premenstrual syndrome and premenstrual dysphoric disorder in Japanese collegiate athletes. J Pediatr Adolesc Gynecol 2015;28:215–218 26028154 10.1016/j.jpag.2014.07.006

[11] Kamagata E, Yamada K Improvements in quality-adjusted life years and cost-utility after pharmacotherapy for premenstrual dysphoric disorder: A retrospective study. Clin Drug Investig 2018;38:49–55 10.1007/s40261-017-0583-3 29032438

[12] Yamada K, Kamagata E Reduction of quality-adjusted life years (QALYs) in patients with premenstrual dysphoric disorder (PMDD). Qual Life Res 2017;26:3069–3073 28674766 10.1007/s11136-017-1642-1

[13] Takeda T, Ueno T, Uchiyama S, Hiramatsu K, Shiina M Relation between premenstrual syndrome and equol-production status. J Obstet Gynaecol Res 2016;42:1575–1580 27352905 10.1111/jog.13073

[14] Henz A, Ferreira CF, Oderich CL, et al. Premenstrual Syndrome Diagnosis: A Comparative Study between the Daily Record of Severity of Problems (DRSP) and the Premenstrual Symptoms Screening Tool (PSST). Rev Bras Ginecol Obstet 2018;40:20–25 29132173 10.1055/s-0037-1608672 PMC10467366

[15] Eisenlohr-Moul TA, Girdler SS, Schmalenberger KM, et al. Toward the reliable diagnosis of DSM-5 premenstrual dysphoric disorder: The Carolina Premenstrual Assessment Scoring System (C-PASS). Am J Psychiatry 2017;174:51–59 27523500 10.1176/appi.ajp.2016.15121510 PMC5205545

[16] Takeda T, Shiina M, Yamada M Translation the Daily Records of Severity of Problems (DRSP) into Japanese and the linguistic validation. [translation from Japanese] Clin Gynecol Obstet 2019;73:807–811

[17] Eremenco S, Pease S, Mann S, Berry P Patient-Reported Outcome (PRO) Consortium translation process: Consensus development of updated best practices. J Patient Rep Outcomes 2017;2:12 29757299 10.1186/s41687-018-0037-6 PMC5934912

[18] Endicott J, Nee J, Harrison W Daily Record of Severity of Problems (DRSP): Reliability and validity. Arch Womens Ment Health 2006;9:41–49 16172836 10.1007/s00737-005-0103-y

[19] Wild D, Grove A, Martin M, et al. Principles of good practice for the translation and cultural adaptation process for patient-reported outcomes (PRO) measures: Report of the ISPOR task force for translation and cultural adaptation. Value Health 2005;8:94–104 15804318 10.1111/j.1524-4733.2005.04054.x

[20] Iwata N, Saito K Psychometric properties of the center for epidemiologic studies depression scale of Japanese workers. Sangyo Igaku 1989;31:20–21 2739098 10.1539/joh1959.31.20

[21] Yoshiko M, Akimoto Y, Ueda K, Toshiko K Development of PMDD scale, and its validity and reliability. [translation from Japanese] J Jpn Soc Psychosom Obstet Gynecol 2009;14:194–201

[22] Miyaoka Y, Akimoto Y, Ueda K, et al. Fulfillment of the premenstrual dysphoric disorder criteria confirmed using a self-rating questionnaire among Japanese women with depressive disorders. Biopsychosoc Med 2011;5:5 21535889 10.1186/1751-0759-5-5 PMC3110105

