# Supplementary material for: Development of a Japanese Version of the Daily Record of Severity of Problems for Diagnosing Premenstrual Syndrome
Source: Womens Health Rep (New Rochelle). 2020 Jan 20;1(1):11–6. doi: 10.1089/whr.2019.0004 (PMC7784737; doi:10.1089/whr.2019.0004)
Supplement: Supplemental data [file Supp_Appendix1.pdf]

Supplementary Data

Supplementary Appendix Table SA1. Patient-Reported Outcomes Consortium Translation Process Steps[S1]

Step No.	Step name	Universal approach
1	Preparation	Obtain permission to translate decide of approach, and Item Definition Table provided. Translation consultants identified for each of the target countries. In-country affiliates identified or back-up option if necessary. Plan for final review and proofreading in the mode to be used in the clinical trial and whether additional text (*e.g.*, error messages and navigational terms) needs translation in addition to the measure itself.
2	Forward translation	Minimum of two forward translations by translators from different target countries if applicable.
3	Reconcilation	Forward translations are reconciled into one translation, with several options to accommodate translation company practices. Universal approach seeks to find a solution that works across target countries. Rationale documented.
4	Back translation	Conduct at least one back translation of the reconciled forward translation. (Back translator to be kept blind to source questionnaire and Item Definition Table.)
5	Revision of reconciled forward translation	Evaluate back translation to assess semantic equivalence and identify issues in the reconciled translation, agree on revisions needed, taking into consideration feedback from multiple target countries, and implement changes.
6	Adaptation of "mother" target language for other countries (country-specific only)	Not applicable.
7	International harmonization	All languages in the project are reviewed for consistency and conceptual equivalence with each other and the original language version.
8	Proofreading	Two or more proofreaders from different target countries check translation, and correct any remaining spelling, diacritical, grammatical or other errors; clinician review is optional. In-country affiliate(s) review translation separately.
9	Cognitive interviewing	Pilot testing and cognitive interviewing conducted in each target country, with a minimum of five participants per language/country who match the target population for as many criteria as reasonably practical. In-person where possible. Testing to be done for target language in each target country in the study associated with that language (*e.g.*, for German, conduct pilot testing in both Germany and Austria). If another relevant country is added in the future, additional cognitive interviews with the universal version in the new country (*e.g.*, Switzerland) need to be conducted.
10	Postcognitive interview review (analysis/revisions)	Review cognitive interview results and compile feedback for translation team resolution. Agree on any revisions to reconciled forward translation identified during cognitive interviews.
11	Final review and documentation (proofreading)	Ensure proposed revision maintains conceptual equivalence and does not threaten international harmonization for future data pooling purposes, implement revisions, proofread revised translations, and document any relevant alternatives in the Item Definition Table. Conduct final proofreading of measure translations (format/layout) for mode(s) of implementation (*e.g.*, screen shots and paper) to identify any mistakes or errors that may impact integrity of data collection.
12	Report	Prepare final summary report documenting development of each translation and providing description of all translation and cultural adaptation decisions.
13	Archiving/record keeping	Documentation to be archived:<br>Qualifications and experience of translation team<br>Documentation of changes made throughout the translation work and rationale for changes<br>Translation certificates<br>Translation report including results of cognitive interviews

Supplementary Reference

S1. Eremenco S, Pease S, Mann S, Berry P. Patient-Reported Outcome (PRO) Consortium translation process: Consensus development of updated best practices. J Patient Rep Outcomes 2017;2:12.
