# Supplementary material for: Development of a Japanese Version of the Daily Record of Severity of Problems for Diagnosing Premenstrual Syndrome
Source: Womens Health Rep (New Rochelle). 2020 Jan 20;1(1):11–6. doi: 10.1089/whr.2019.0004 (PMC7784737; doi:10.1089/whr.2019.0004)
Supplement: Supplemental data [file Supp_Appendix2.pdf]

Supplementary Appendix Table SA2. Japanese Translation and Adaptation Process of Daily Record of Severity of Problems

	Original DRSP	Forward translation Step 2	Forward translation reconciled Step 3	Back translation 1 Step 4	Back translation 2 Step 4	Revision of forward translation Steps 5, 6
1	Felt depressed, sad, down, or blue	憂鬱な気分、または落ち込みやすい	気分が沈む、悲しくなる、落ち込む、気が滅入る	Feeling down, sad, upset, and depressed	Feel down, sad, melancholic, and depressed	気分が沈んだ、悲しくなった、落ち込んだ、気が滅入った
2	Felt hopeless	絶望的な気分	希望が持てない	Feeling hopeless	Feel hopeless	希望が持てなかった
3	Felt worthless or guilty	自分には価値がないように思う、または罪悪感がある	自分に価値がない、または自分が悪いと感じる	Feeling worthless or at fault	Feel worthless or sinful	自分が役に立たない、申し訳ないと感じた
4	Felt anxious, tense, keyed up, or on edge	不安、または張り詰めた気持ち	不安と緊張を感じる、ピリピリしている、ぎりぎりな感じ	Feeling anxious and tense, irritated, and barely making it	Feel uneasy, nervous, tense, and strained	不安と緊張を感じた、ピリピリしていた、ぎりぎりな感じだった
5	Had mood swings (*e.g.*, suddenly felt sad or tearful)	気分のアップダウンが大きい（急に悲しくなったり涙が出たりする）	気分に波がある（急に悲しくなったり涙が出たりする）	Mood swings (suddenly feeling sad or becoming teary)	Mood swings (sudden sadness or tearfulness)	気分に波があった（急に悲しくなったり涙が出たりした）
6	Was more sensitive to rejection or feelings were more easily hurt	否定されているように感じやすい、傷つきやすい	断られることに敏感になり、傷つきやすい	Sensitive to rejection, easily hurt	Fear rejection, easily hurt	断られることに敏感になり、傷つきやすかった
7	Felt angry, irritable	イライラしやすい	イライラして怒りっぽい	Irritated and angry	Frustrated, short-tempered	イライラして怒りっぽかった
8	Had conflicts or problems with people	人と衝突する	人と衝突したり、揉めたりする	Conflicting with others, getting into trouble	Clash or argue with others	人と衝突したり、うまくいかなかったりした。
9	Had less interest in usual activities (*e.g.*, work, school, friends, and hobbies)	興味がわかない（仕事、学校、友達、趣味）	普段興味がある活動に興味がもてなくなる（仕事、学校、友達、趣味のことなど）	Loss of interest in daily activities (work, school, friends, hobbies, etc.)	Lost interest in normal interests (work, school, friends, and hobbies)	普段している活動に興味が持てなくなった（仕事、学校、友達、趣味のことなど）
10	Had difficulty concentrating	集中しにくい	集中しにくい	Difficulty concentrating	Have trouble concentrating	集中しにくかった
11	Felt lethargic, tired, fatigued, or had a lack of energy	疲れやすい、だるい	疲れやすい、だるい、元気が出ない、しんどい	Feeling tired or dull, not feeling energized, exhausted	Easily tired, languid, enervated, and lethargic	疲れやすかった、だるかった、元気が出なかった
12	Had increased appetite or overate	食欲亢進または食べ過ぎる	食欲が増す、食べ過ぎる	Increased appetite or overeating	Insatiable appetite or overeat	食欲が増した、食べ過ぎた
13	Had cravings for specific foods	特定の食べ物を無茶食いした	同じものばかり食べたくてしかたない	Craving for the same foods	Irrepressible desire to eat a single food	特定のものを食べたくてしかたなかった
14	Slept more, took naps, found it hard to get up when intended	過眠、おきられない	寝すぎる、居眠りする、起きようとしても起きられない	Sleeping too much, falling asleep during the day, or difficulty waking up	Oversleep, doze off, or unable to get up	寝すぎた、居眠りした、起きづらかった
15	Had trouble getting to sleep or staying asleep	寝つきがわるい、または中途覚醒	寝つきが悪い、途中で目がさめる	Difficulty falling asleep or waking up in the middle of the night	Have trouble falling or staying asleep	寝つきが悪かった、途中で目がさめた
16	Felt overwhelmed or that I could not cope	圧倒される感じ	どうにもならないと感じる	Feeling overwhelmed	Feel helpless	手に負えない、どうにもならないと感じた
17	Felt out of control	コントロールできない	自分をコントロールできない	Feeling out of control	Lose self-control	自分をうまく抑えられなかった
18	Had breast tenderness	乳房の痛み	乳房の痛み	Breast pain	Feel breast pain	乳房の痛みがあった
19	Had breast swelling, felt bloated, or had weight gain	乳房の張り、体のむくみ	乳房の張り、お腹の張り、体重増加	Breast tenderness, abdominal bloating, or weight gain	Feel chest or stomach tension or weight gain	乳房の張り、お腹の張り、体重増加があった
20	Had headache	頭痛	頭痛	Headache	Headache	頭痛があった

(*continued*)

Supplementary Appendix Table SA2. Continued

	Original DRSP	Forward translation Step 2	Forward translation reconciled Step 3	Back translation 1 Step 4	Back translation 2 Step 4	Revision of forward translation Steps 5, 6
21	Had joint or muscle pain	関節痛または筋肉痛	関節痛または筋肉痛	Joints or muscle pain	Feel joint or muscle pain	関節痛または筋肉痛があった
22	At work, school, home, or in daily routine, at least one of the problems already noted caused reduced productivity or inefficiency	上記のいずれかの症状のために、職場、学校、家庭、日常生活において、生産性や効率が落ちた	上記のいずれかの症状のために、職場、学校、家庭、日常生活において、生産性や効率が落ちた	Decline in productivity and efficiency in the workplace, school, home, and daily life due to any of the symptoms already mentioned	Lower productivity or efficiency due to the mentioned	上記のいずれかの症状のために、職場、学校、家庭、日常生活において、生産性や効率が落ちた
23	At least one of the problems already noted interfered with hobbies or social activities (*e.g.*, avoided or did less)	上記のいずれかの症状のために趣味や人付き合いができなかった	上記のいずれかの症状のために趣味や人付き合いができなかった	Relationships and hobbies affected by any of the symptoms already mentioned	Lost interests or friendships due to the mentioned	上記のいずれかの症状のために趣味や外での活動に支障があった。
24	At least one of the problems already noted interfered with relationships with others	上記のいずれかの症状のために対人関係が悪くなった	上記のいずれかの症状のために対人関係が悪くなった	Social relationships affected by any of the symptoms already mentioned	Worsened human relationships due to that mentioned	上記のいずれかの症状のために対人関係が悪くなった
	Record the score for each item on each day using the following scale of 1 to 6	次のスケールを用いて毎日各項目にスコアをつけてください。	次のスケールを用いて毎日各項目にスコアをつけてください。	Using the following scale, please record the daily score for each item	Rank yourself daily using the following scale	次のスケールを用いて毎日夕方に記録してください。今日1日を振り返って、以下の症状がありましたか?
	1 = not at all<br>2 = minimal<br>3 = mild<br>4 = moderate<br>5 = severe<br>6 = extreme	1. 全くない<br>2. ごくわずか<br>3. 軽度<br>4. 中程度<br>5. 強い<br>6. 非常に強い	1. 全くない<br>2. ごくわずか<br>3. 軽度<br>4. 中程度<br>5. 強い<br>6. 非常に強い	1. None<br>2. Minimal<br>3. Mild<br>4. Moderate<br>5. Severe<br>6. Extremely severe	1. None<br>2. Very little<br>3. Mild/slight/occasionally<br>4. Moderate/sometimes<br>5. Strong<br>6. Very strong	1 全くない<br>2 ごくわずか<br>3 軽度<br>4 中程度<br>5 強い<br>6 非常に強い

DRSP, daily record of severity of problems.
