# Supplementary material for: Development of a Japanese Version of the Daily Record of Severity of Problems for Diagnosing Premenstrual Syndrome
Source: Womens Health Rep (New Rochelle). 2020 Jan 20;1(1):11–6. doi: 10.1089/whr.2019.0004 (PMC7784737; doi:10.1089/whr.2019.0004)
Supplement: Supplemental data [file Supp_Appendix3.docx]

Appendix3. DRSP Sheet in Japanese
